# Tanshinone IIA Sodium Sulfonate Attenuates LPS-Induced Intestinal Injury in Mice

**DOI:** 10.1155/2018/9867150

**Published:** 2018-03-08

**Authors:** Xin-Jing Yang, Jin-Xian Qian, Yao Wei, Qiang Guo, Jun Jin, Xue Sun, Sheng-Lan Liu, Chun-Fang Xu, Guo-Xing Zhang

**Affiliations:** ^1^Department of Intensive Care Unit, The First Affiliated Hospital, Soochow University, 188 Shi-Zi Road, Suzhou 215006, China; ^2^Department of Emergency, Affiliated Suzhou Hospital of Nanjing Medical University, Suzhou Municipal Hospital, Suzhou, China; ^3^Department of Gastroenterology, The First Affiliated Hospital, Soochow University, 188 Shi-Zi Road, Suzhou 215006, China; ^4^Department of Physiology and Neuroscience, Medical College of Soochow University, 199 Ren-Ai Road, Dushu Lake Campus, Suzhou Industrial Park, Suzhou 215123, China

## Abstract

**Background:**

Tanshinone IIA sodium sulfonate (TSS) is known to possess anti-inflammatory effects and has exhibited protective effects in various inflammatory conditions; however, its role in lipopolysaccharide- (LPS-) induced intestinal injury is still unknown.

**Objective:**

The present study is designed to explore the role and possible mechanism of TSS in LPS-induced intestinal injury.

**Methods:**

Male C57BL/6J mice, challenged with intraperitoneal LPS injection, were treated with or without TSS 0.5 h prior to LPS exposure. At 1, 6, and 12 h after LPS injection, mice were sacrificed, and the small intestine was excised. The intestinal tissue injury was analyzed by HE staining. Inflammatory factors (TNF-*α*, IL-1*β*, and IL-6) in the intestinal tissue were examined by ELISA and RT-PCR. In addition, expressions of autophagy markers (microtubule-associated light chain 3 (LC3) and Beclin-1) were detected by western blot and RT-PCR. A number of autophagosomes were also observed under electron microscopy.

**Results:**

TSS treatment significantly attenuated small intestinal epithelium injury induced by LPS. LPS-induced release of inflammatory mediators, including TNF-*α*, IL-1*β*, and IL-6, were markedly inhibited by TSS. Furthermore, TSS treatment could effectively upregulate LPS-induced decrease of autophagy levels, as evidenced by the increased expression of LC3 and Beclin-1, and more autophagosomes.

**Conclusion:**

The protective effect of TSS on LPS-induced small intestinal injury may be attributed to the inhibition of inflammatory factors and promotion of autophagy levels. The present study may provide novel insight into the molecular mechanisms of TSS on the treatment of intestinal injury.

## 1. Introduction

Sepsis is a complicated clinical syndrome mainly due to a harmful host inflammatory response to infection. Despite advancements in understanding the pathophysiology of sepsis, clinical outcomes are still worrisome, and the mortality rate remains high among the patients [1]. Bacterial infection is one of the major causes of sepsis. Gram-negative bacteria induced infection via lipopolysaccharide (LPS, the major constituent of the outer membrane) binding to Toll-like receptor 4 (TLR4) to produce pleiotropic inflammatory reaction [2, 3]. The increase of proinflammatory mediators such as interleukin- (IL-) 1*β* and tumor necrosis factor-*α* (TNF-*α*) results in the activation of leukocytes and macrophages which were sequestrated in the tissue and contribute to vascular damage and the development of systemic inflammatory reaction [4, 5]. Clinically, numerous strategies have been explored to inhibit the development of the bacterial infection by inhibition of bacterial growth or of inflammatory reaction. However, it is still necessary to discover novel therapy for the clinical patients in response to increased bacterial infection and sepsis.

Autophagy is a bulk intracellular degradation system that delivers cytoplasmic proteins and organelles to lysosomes for degradation and recycling [6]. Under normal conditions, autophagy plays an important role in development, cell survival, and differentiation [7, 8]; however, it may also participate in pathological processes. Autophagy has been reported to be involved in inflammatory diseases. An increase in the degree of inflammation in the tissues that led to impaired clearance of apoptotic cells was observed in Atg5-deficient embryos [9]. In addition, the liver tissue of Beclin-1 mutant mice showed not only enhanced apoptosis and tissue damage but also increased inflammation, which lead to steatohepatitis and hepatocellular carcinoma [10]. Thus, impaired autophagy may result in progression of inflammation, for example, via the accumulation of aggregated proteins, suggesting that autophagy may be associated with inflammatory responses. Nevertheless, whether the process of autophagy is generally beneficial or harmful in the intestinal inflammatory response is still not well documented.

Tanshinone IIA sodium sulfonate (TSS), the major bioactive compound of *Salvia miltiorrhiza*, has been widely used in clinical treatment for thousands of years in China for the treatment of various microcirculatory disturbance-related diseases [11]. Recently, its effects of anti-inflammation [12], antitumor [13, 14], and antibacteria [15] have been widely investigated. Our recent study also demonstrated that TSS ameliorates ischemia-reperfusion-induced myocardial injury and improves cardiac function via reducing inflammation and apoptosis, while enhancing autophagy [16]. However, there is still no observation of the effects of TSS on intestinal inflammation.

In the present study, we examine whether TSS possesses therapeutic effects on LPS-induced intestinal injury and explore the possible mechanism involved.

## 2. Materials and Methods

### 2.1. Materials

**Figure 1:**
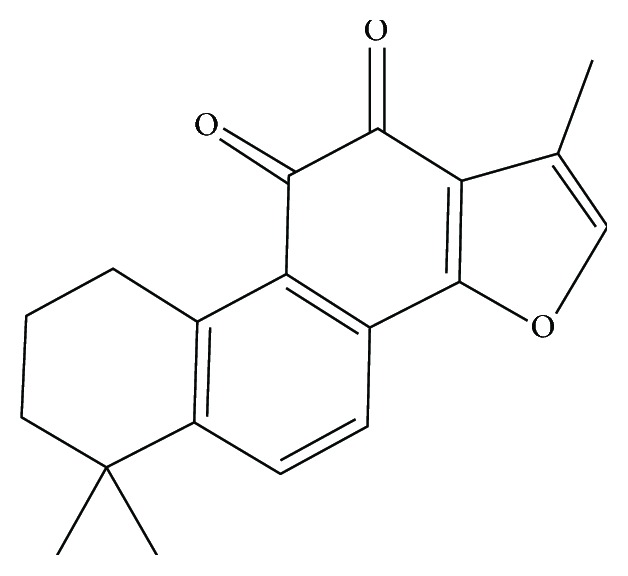
Chemical structure of TSS.

Chemical tanshinone IIA sodium sulfonate (TSS, purity is 99%) was purchased from the National Institute for the Control of Pharmaceutical and Biological Products (Beijing, China). The structure of TIIA is shown in Figure 1. Enzyme-linked immunosorbent assay (ELISA) kits for TNF-*α*, IL-6, and IL-1*β* were obtained from R&D Systems (Minneapolis, MN, USA). Monoclonal *β*-actin antibody, *Escherichia coli* endotoxin LPS (O55:B5), Evans blue dye, and all the other reagents were obtained from Sigma-Aldrich Inc. (St. Louis, MO, USA). The purity of all chemical reagents was at least in analytical grade.

### 2.2. Animals

Six-week-old male C57BL/6J mice (body weight 20 ± 2 g) were purchased from Shanghai Animal Laboratory Center. The mice were housed under optimal conditions with standard hygiene, kept at a temperature of 25°C with a12/12 light/dark cycle, and fed with standard mice chow and water ad libitum. The mice were randomized into three groups: (1) control group, mice received saline administration (*n* = 10); (2) LPS group, mice received LPS 30 mg/kg (*n* = 10); and (3) LPS + TSS group, mice were pretreated with TSS (30 mg/kg) [16], and 0.5 h later received LPS (30 mg/kg, *n* = 10). In all groups, administration was via intraperitoneal injection. At 1, 6, and 12 h after LPS injection, the mice were sacrificed, and the jejunum and ileum were excised for further investigation. The experiments were performed in accordance with the National Institutes of Health Guidelines for the Use of Laboratory Animals (NIH, publication number 85-23, revised 1996.), which were approved and performed according to guidelines for the care and use of animals established by Soochow University.

### 2.3. Histology Studies

The regions of mouse jejunum and ileum were washed with PBS and fixed in 4% formaldehyde for 1 h at 4°C. The tissues were dehydrated by gradually soaking them in alcohol and xylene and then embedded in paraffin. The paraffin-embedded specimens were cut into 5 *μ*m sections, stained with hematoxylin-eosin (H&E), and viewed with a digital light microscope (Olympus, Tokyo, Japan). The length of intestine villus was measured by NIH image.

### 2.4. ELISA Cytokine Determination

The jejunum samples were weighed (200 mg/ml) and homogenized with Tris-EDTA buffer (10 mM Tris-HCl and 1 mM EDTA, pH 7.4; 0.05% sodium azide; 1% Tween-80; and protease inhibitor cocktail), centrifuged at 11,000 ×g for 10 min at 4°C, and supernatant was collected. Inflammatory factors (TNF-*α*, IL-1*β*, and IL-6) from tissue homogenates were measured using the cytometric bead array mouse inflammation kit (BD Pharmingen) according to the manufacturer's instruction.

### 2.5. Real-Time Quantitative PCR

Total RNA was isolated from jejunum tissues using the RNeasy Mini Kit (Qiagen, Valencia, CA, USA). cDNA was synthesized by reverse transcription from 50 ng total RNA using a cDNA Reverse Transcriptase Kit (TaKaRa, Tokyo, Japan). Real-time quantitative PCR was carried out in a 384-well plate using the ABI Prism 7900HT Sequence Detection System (Thermo Fisher Scientific) with the following conditions: 95°C, 10 min (95°C, 10 s; 60°C, 10 s; and 72°C, 15 s) for 40 cycles. The primer pairs for TNF-*α* cDNA were 5′-GCCAGGAGGGAGAACAGAAACTC-3′ (forward) and 5′-GGCCAGTGAGTGAAAGGGACA-3′ (reverse). The primer pairs for IL-1*β* cDNA were 5′-TCCAGGATGAGGACATGAGCAC-3′ (forward) and 5′-GAACGTCACACACCAGCAGGTTA-3′ (reverse). The primer pairs for IL-6 cDNA were 5′-CCACTTCACAAGTCGGAGGCTTA-3′ (forward) and 5′-CCAGTTTGGTAGCATCCATCATTTC-3′ (reverse). The primer pairs for Beclin-1 cDNA were 5′-ATGGAGGGGTCTAAGGCGTC (forward) and 5′-TGGGCTGTGGTAAGTAATGGA (reverse). The primer pairs for LC3 cDNA were 5 CGCTTGCAGCTCAATGCTAAC-3′ (forward) and 5′-TCTCTCACTCTCGTACACTTCG-3′ (reverse). The primer pairs for GAPDH cDNA were 5′-AAATGGTGAAGGTCGGTGTGAAC-3′ (forward) and 5′-CAACAATCTCCACTTTGCCACTG-3′ (reverse). TNF-*α*, IL-1*β*, IL-6, Beclin-1, and LC3 mRNAs were normalized to GAPDH mRNA.

### 2.6. Western Blotting Analysis

The jejunum tissues were homogenized with radioimmunoprecipitation assay (RIPA) buffer (50 mm Tris, pH 7.0; 150 mM NaCl; and 1% Triton-X-100) containing phenylmethanesulfonyl fluoride (R&D Systems Inc., Minneapolis, US). Homogenates were centrifuged at 12,000 ×g for 10 min at 4°C. Sample protein was separated by SDS-PAGE and transferred to PVDF membranes (Hybond TM-ECL; Amersham Pharmacia Biotech Inc.). The membranes were blocked in 5% nonfat milk in PBS and 0.1% Tween-20 at room temperature. The blots were then incubated with primary antibody: anti-Beclin-1 antibody (1 : 1000, Abcam Inc.), anti-LC3 (1 : 1000, Abcam Inc.), or anti-GAPDH (Santa Cruz Biotech Inc.). Then, the membranes were incubated for 1 hour with a secondary antibody (HRP-conjugated antirabbit Ig-G, 1 : 2000). Excess antibody was washed off with TBS-T three times (15 minutes each) before incubation enhanced chemiluminescent reagent (ECL, R&D Systems Inc., Minneapolis, USA) for 1 min. Subsequently, the membrane was exposed to X-ray film. Immunoreactive bands were detected by the analysis of X-ray films using the software of Image J. The quantity of target proteins is normalized by GAPDH expression.

### 2.7. Electron Microscopy

The jejunum tissues were fixed with a fixation solution (2% paraformaldehyde and 2% glutaraldehyde in 0.05 M sodium cacodylate buffer, pH 7.2) after the mice were perfused with cold PBS. Fixed samples were embedded in Spur's resin (14300; Electron Microscopy Sciences, Hatfield, PA, USA), and thin sections (80 nm) were cut and processed for transmission electron microscopy. After dehydration, the thin sections were stained with uranyl acetate and lead citrate and observed under a JEM1011CX electron microscope with accelerating voltage of 100 kV (H7650; Hitachi, Tokyo, Japan). Images were acquired from a randomly selected pool of 5–8 fields under each condition.

### 2.8. Statistical Analysis

The SPSS 18.0 software was used for statistical analysis. Data were presented as the mean ± SEM. Grouped data were analyzed using a one-way analysis of variance followed by the Student-Newman-Keuls test. A *P* value < 0.05 was considered to be statistically significant.

## 3. Results

### 3.1. TSS Improved LPS-Induced Intestine Histopathologic Changes

**Figure 2:**
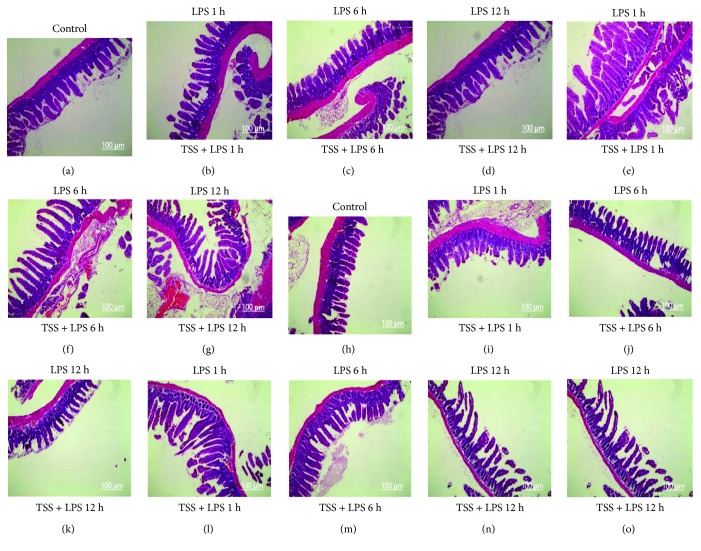
Effects of TSS on small intestinal injury in LPS-induced mice (H&E stain, ×100). Architecture of epithelium and wall of jejunum (a) and ileum (h) from control mice. Architecture of epithelium and wall of jejunum (b, c, d) and ileum (i, j, k) from LPS-injected mice for 1, 6, and 12 hours. Architecture of epithelium and wall of jejunum (e, f, g) and ileum (l, m, n, o) from LPS-injected mice for 1, 6, and 12 hours pretreated with TSS.

Histological analysis showed the jejunum and ileum from control mice had the normal architecture of the intestinal epithelium and wall (Figures 2(a) and 2(h)), while LPS induced severe edema and sloughing of the villus tips, as well as infiltration of inflammatory cells into the mucosa at all observed time points (Figures 2(b)–2(d), 2(i)–2(k)). In contrast, TSS treatment significantly reduced the edema and inflammatory cell infiltration of the jejunum and ileum compared with the LPS group at all observed time points (Figures 2(e)–2(g), 2(l)–2(o), *P* < 0.05). These results at least suggest that TSS could ameliorate LPS-induced intestine histopathologic changes.

**Figure 3:**
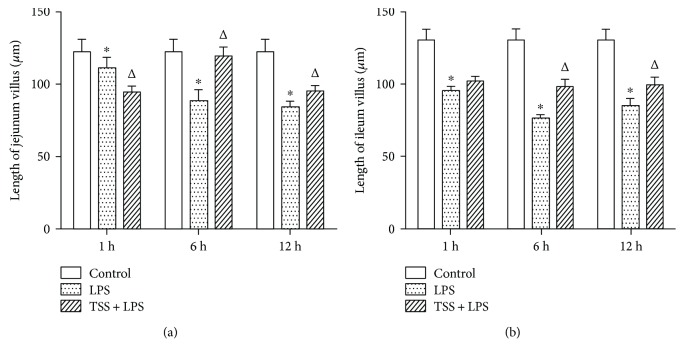
Effects of TSS on LPS-induced shortening of jejunum and ileum villus. The length of jejunum (a) and ileum (b) villus in each group at different time points. LPS: lipopolysaccharide; TSS: tanshinone IIA sodium sulfonate. Blank column indicates control group; dot column indicates LPS treatment at all observed time points; diagonal column indicates TSS pretreatment at all observed time points; *n* = 10 in each group. Each bar presents the mean ± SEM. ^∗^*P* < 0.05 versus control group. Δ*P* < 0.05 versus LPS group.

The length of jejunum and ileum villus was time-dependently shortened in response to LPS stimulation and peaked at 6 h compared with control group (Figures 3(a) and 3(b), *P* < 0.05). TSS treatment at 1 h after LPS stimulation had no marked effect on the shortening of jejunum and ileum villus; however, from 6 h to 12 h, TSS treatment significantly inhibited the reduction of villus length compared with LPS group. (Figures 3(a) and 3(b), *P* < 0.05). These data strongly suggested that TSS could inhibit LPS-induced intestine damage.

### 3.2. TSS Attenuated LPS-Induced Intestine Inflammatory Response

**Figure 4:**
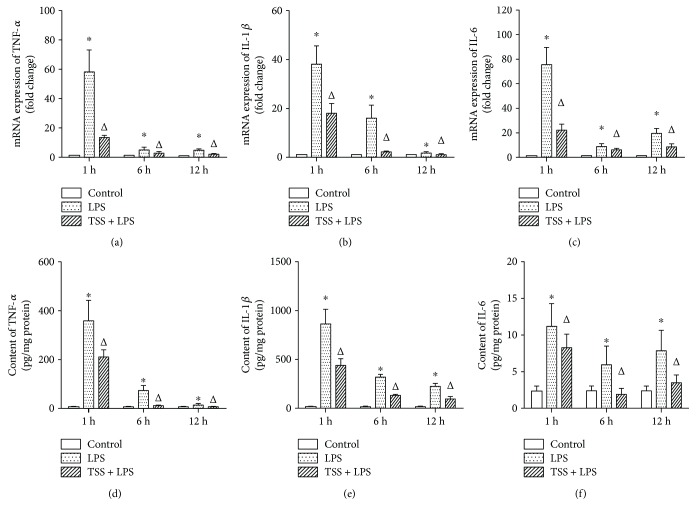
Expression TNF*α*, IL-1*β*, and IL-6 in jejunum tissue. (a) mRNA of TNF-*α* expression. (b) mRNA of IL-1*β* expression. (c) mRNA of IL-6 expression. (d) Protein content of TNF-*α*. (e) Protein content of IL-1*β*. (f) Protein content of IL-6. LPS: lipopolysaccharide; TSS: tanshinone IIA sodium sulfonate. Blank column indicates control group; dot column indicates LPS treatment at all observed time points; diagonal column indicates TSS pretreatment at all observed time points; *n* = 10 in each group. Each bar presents the mean ± SEM. ^∗^*P* < 0.05 versus control group. Δ*P* < 0.05 versus LPS group.

In the present study, inflammatory cytokines were measured to assess intestine inflammatory response. We measured the levels of inflammatory cytokines such as TNF-*α*, IL-6, and IL-1*β* by RT-PCR and ELISA. After LPS stimulation, both the mRNA and protein expressions of TNF-*α*, IL-6, and IL-1*β* were markedly increased at all observed time points compared to those of control group, which were time-dependently decreased (Figures 4(a)–4(f), *P* < 0.05). TSS treatment inhibited not only the protein expressions of TNF-*α*, IL-6, and IL-1*β* but also the mRNA expressions at all observed time points (Figures 4(a)–4(f), *P* < 0.05). These results indicate that LPS-induced release of inflammatory cytokines is time dependent and mainly occurs at early time points. In addition, these data also confirm that TSS has anti-inflammatory effects in response to LPS challenge.

### 3.3. TSS Upregulates LPS-Induced Decrease of Intestinal Autophagy

**Figure 5:**
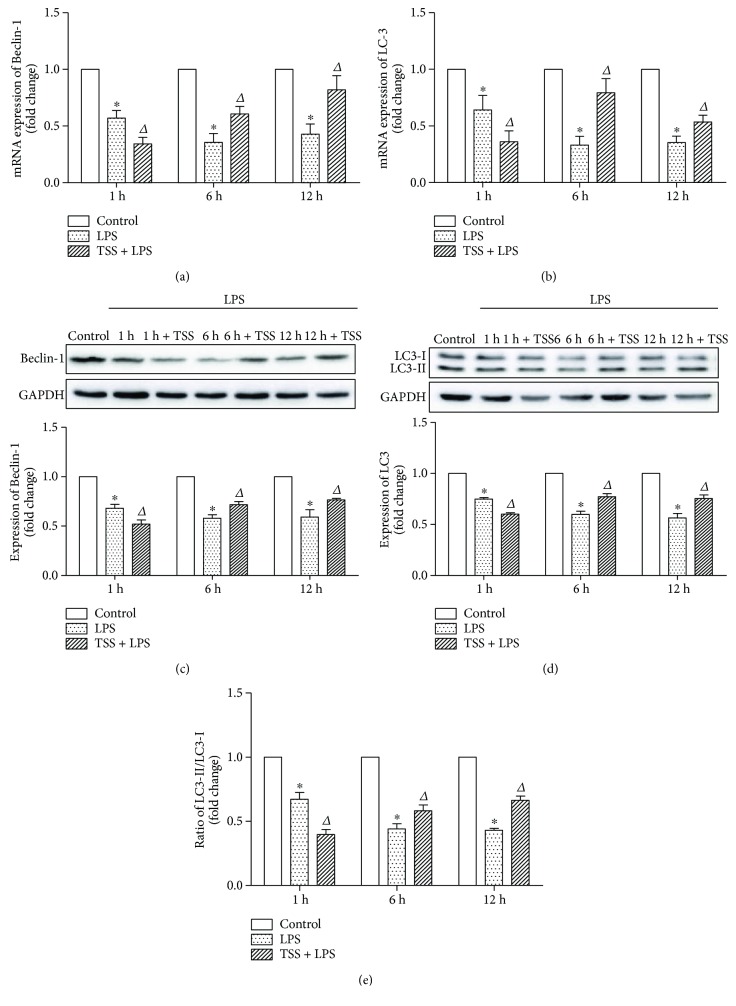
Expression of autophagic markers Beclin-1 and LC3. (a) mRNA expression of Beclin-1 in each group. (b) mRNA expression of LC3 in each group. (c) Protein expression of Beclin-1 in each group; the upper part is the representative blots of Beclin-1 and GAPDH; the lower part is the densitometric analysis of Beclin-1 expression normalized to GAPDH. (d) Protein expression of LC3 in each group. The upper part is the representative blots of LC3; the lower part is the densitometric analysis of LC3 expression normalized to GAPDH. (e) Ratio of LC3-II to LC3-I in each group. LPS: lipopolysaccharide; TSS: tanshinone IIA sodium sulfonate. Blank column indicates control group; dot column indicates LPS treatment at all observed time points; diagonal column indicates TSS pretreatment at all observed time points; *n* = 10 in each group. Each bar presents the mean ± SEM. ^∗^*P* < 0.05 versus control group. Δ*P* < 0.05 versus LPS group.

To investigate the effects of TSS on tissue autophagy levels, the expressions of Beclin-1 and the ratio of LC3-II to LC3-I were determined by RT-PCR and western blot analysis. Our results demonstrated that LPS stimulation decreased the tissue autophagy levels as evidenced by reductions of both mRNA and protein expressions of Beclin-1 and LC3, also the ratio of LC3-II to LC3-I compared with control group as early as from 1 hour (Figures 5(a)–5(e), *P* < 0.05). Pretreatment with TSS at early time point (1 hour) showed more reduction of autophagy levels; however, from 6 hours to 12 hours, TSS treatment significantly increased the mRNA and protein expressions of Beclin-1 and LC3, also the ratio of LC3-II to LC3-I, compared with LPS group (Figures 5(a)–5(e), *P* < 0.05). These results indicate that LPS-induced tissue injury may be partially due to the destruction of cell self-repairing function (autophagy); it also suggests that TSS may exert its effects via restoring the tissue autophagy levels.

**Figure 6:**
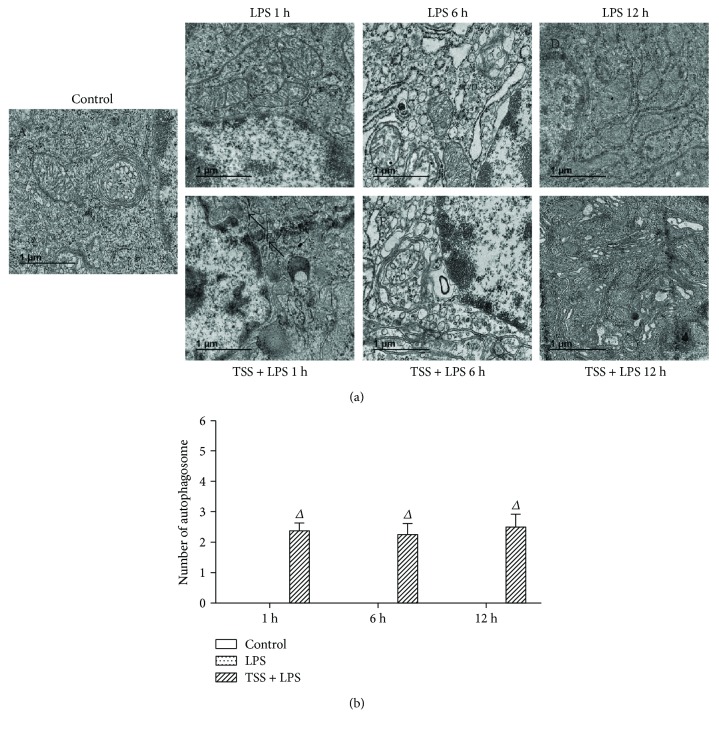
Autophagosome formation in each group. Representative transmission electron microscope images of each group. (a) A: the control group showed the normal jejunum electron microscopy images; B, C, D: electron microscopy images in jejunum endothelium of LPS-induced mice; E, F, G: the electron microscopy images changed in the jejunum epithelial cells when treated with TSS and LPS. (b) Random cells were chosen to determine the number of autophagosomes/cell for each treatment condition. The black arrowheads indicate membrane-bound vacuoles that are characteristic of autophagosomes. LPS: lipopolysaccharide; TSS: tanshinone IIA sodium sulfonate. Blank column indicates control group; dot column indicates LPS treatment at all observed time points; diagonal column indicates TSS pretreatment at all observed time points; *n* = 5 in each group. Each bar presents the mean ± SEM. Δ*P* < 0.05 versus LPS group.

In addition, by transmission electron microscope observation, we confirmed morphological changes in the jejunum and excess vacuolization in the epithelial cells (Figure 6(a)). Results showed that in the LPS group, any double or multiple membrane structures engulfed with cell organelle could not be observed, which are key features of autophagy at all time points; however, in the TSS treatment group, these structures could be found at all time points (Figure 6(b)). These results further confirmed that TSS could upregulate intestinal autophagy levels, which may contribute to the protection effects in response to LPS-induced injury.

## 4. Discussion

The gut, regarded as initiating multiple organ failure and its potential pathogenicity, has been given more attention. The gut became a “cytokine-releasing organ” and can amplify the early systemic inflammatory response syndrome. This early gut injury has been considered to be the “motor” that drives sepsis-induced multiple organ failure [17]. In the present study, *Escherichia coli* LPS, a potent endotoxin, was injected into the mice to establish intestinal injury model. We observed that LPS induced severe edema and sloughing of the villus tips, as well as infiltration of inflammatory cells into the mucosa. At 1 h after LPS exposure, the expressions of inflammatory cytokines, TNF-*α*, IL-6, and IL-1*β*, were markedly increased compared to control group, suggesting the quick response of inflammation in gut, which may contribute to the gut injury and subsequent systemic organ inflammation.

Danshen, one herbal drug derived from the dried root or rhizome of *Salvia miltiorrhiza* Bunge, has been used clinically to manage many diseases, such as angina pectoris, myocardial infarction, and stroke [18–20]. TSS, one of the key components of Danshen, has been reported to possess the majority property of Danshen with few side effects [21]. Previous study has demonstrated that TSS inhibits the production of proinflammatory mediators such as NO, TNF-*α*, IL-1*β*, and IL-6 by the inhibition of NF-*κ*B activation in RAW 264.7 cells stimulated with LPS [22], which has been demonstrated to be mediated by estrogen receptor activation [23]. Our present results for the first time showed that TSS treatment therapeutically inhibited LPS-induced intestinal injury in mice, as demonstrated by the improvement in intestine morphology. We next examined whether TSS could attenuate LPS-induced inflammatory responses in the intestine. Our results clearly demonstrated that the expression of inflammatory cytokines, such as TNF-*α*, IL-6, and IL-1*β*, were inhibited by TSS in response to LPS stimulation. These results indicate that TSS may attenuate LPS-induced intestinal damage via inhibition of inflammatory response.

There is a growing interest in translating autophagy studies into better understanding of human diseases [24]. However, the in vivo roles of autophagy in relation to organ pathology are not well understood. Recent accumulating evidence showed a close association between autophagy in the intestinal epithelial cells and inflammatory bowel diseases (IBDs); indeed, single-nucleotide polymorphisms (SNPs) within two autophagy-related genes, autophagy-related 16-like 1(ATG16L1) and the immunity-related GTPase family M (IRGM), are important loci for IBD pathogenesis [25, 26]. Dysfunction of these autophagy-related proteins is associated with survival of intracellular bacteria, proinflammatory cytokine secretion, and colitis onset [25, 27]. Moreover, existing evidence showed that autophagy plays an important role in intestinal epithelial with endotoxin-induced inflammatory responses [28, 29]. Therefore, we investigate the effects of TSS on autophagy levels in LPS-induced intestinal injury model, which has never been reported until now.

Under normal physiological conditions, low levels of autophagy are maintained that play a vital role in host defenses against microbial infection [30]. Accumulating evidence from in vitro and in vivo studies suggests that autophagy plays a protective role in sepsis [31, 32], although contradictory findings have been reported [33]. However, the present opinion is that both cell and animal models suggest that autophagy is induced in the early stage of sepsis, and the late-stage exhaustion of autophagic activity is associated with inflammatory dysregulation, histological changes, mitochondrial dysfunction, and apoptosis [34]. Thereafter, the role of autophagy in response to sepsis is time dependent. Our present study clearly demonstrated the decrease of autophagy levels as early as 1 hour after LPS stimulation, indicating that intestine tissue is more sensitive to LPS to initiate original protection mechanism resulting in quick exhaustion of autophagic activity. This could explain why our results show the discrepancy of autophagosome formation and molecular markers of autophagy (Beclin-1, LC3) at 1 h observed time points. Nonetheless, dysfunctions in the autophagy pathway have been linked to the pathogenic conditions involving autophagic cell death. Recently, it was discovered that probiotic *Bacillus amyloliquefaciens* SC06-induced autophagy plays a role in the elimination of intracellular bacteria when RAW 264.7 cells were challenged with *E*. *coli* [28]. And ghrelin could enhance the autophagy of intestinal epithelial cells in rats with sepsis and protect the small intestinal epithelium against sepsis-induced injury [35]. Based on the abovementioned literatures, we hypothesize that the regulation of autophagic balance could be manipulated as a mechanism of LPS-related intestinal inflammation injury. Fortunately, our results strongly support that TSS could upregulate expressions of autophagic markers, Beclin-1 and LC3, from 6 h to 12 h in contrast to LPS group. It reveals a novel mechanism for the protective effects of TSS in LPS-induced intestinal injury model, which may occur via the activation of self-repair mechanisms to rescue damaged cells. At present, the mechanisms by which TSS regulates autophagy have not been clearly characterized; however, several studies have indicated the involvement of nuclear factor-kappa B (NF-*κ*B). For example, activated NF-*κ*B may repress the autophagy of Ewing's sarcoma cells [36]. In contrast, autophagy could inhibit NF-*κ*B activation to reduce endotoxin-induced inflammatory responses in the intestinal epithelium [37].

In summary, our study provides evidence for the first time that TSS inhibits LPS-induced intestinal injury by the depression of inflammation and upregulation of autophagy in mice. Although further studies need to be performed in the clinic, our present results provide a solid basis for employing TSS in the treatment of intestinal injury.
